# Unerwünschte Wirkungen von Januskinase‐Inhibitoren mit Relevanz für den dermatologischen Klinik‐ und Praxisalltag

**DOI:** 10.1111/ddg.15796_g

**Published:** 2025-09-15

**Authors:** Anika Rajput Khokhar, Kamran Ghoreschi, Julia Huynh

**Affiliations:** ^1^ Klinik für Dermatologie Venerologie und Allergologie Charité – Universitätsmedizin Berlin corporate member of Freie Universität Berlin and Humboldt‐Universität zu Berlin

**Keywords:** Atopische Dermatitis, Januskinaseinhibitor, Nebenwirkung, Pharmakologie, Psoriasis, Atopic dermatitis, Janus kinase inhibitor, Pharmacology, Psoriasis, Side effect

## Abstract

Seit 2011 in den USA und 2012 in der EU eingesetzt, etablieren sich Januskinase‐Inhibitoren (JAKi) zunehmend in der Therapie dermatologischer Erkrankungen wie atopische Dermatitis, Psoriasis, Psoriasis‐Arthritis, Alopecia areata, chronisches Handekzem und Vitiligo. Für den sicheren und effektiven Einsatz sind Kenntnisse über ihren Wirkmechanismus und potenzielle Nebenwirkungen notwendig. Ihre kurze Halbwertszeit erfordert eine tägliche Einnahme, ermöglicht eine gute Steuerbarkeit und wird aufgrund des schnellen Wirkungseintritts sowie des Verzichts auf subkutane oder intravenöse Injektionen von vielen Patienten geschätzt.

Häufige Nebenwirkungen sind Infekte der oberen Atemwege sowie Varizella‐ Zoster‐Virus (VZV)‐Reaktivierungen. Selten können schwere Infektionen auftreten, manchmal mit problematischem Verlauf. Ein erhöhtes Risiko für kardiovaskuläre Ereignisse wurde bei bestimmten JAKi beschrieben, weshalb bei Patienten mit kardiovaskulärem Risiko alternative Therapien bevorzugt werden sollten. In Studien zur rheumatoiden Arthritis wurde unter Tofacitinib eine erhöhte Inzidenz von Malignomen (Bronchialkarzinom, Lymphom) festgestellt. Zudem wurden aggressivere Verläufe epithelialer Hauttumoren unter JAKi beobachtet. Tierstudien weisen auf teratogene Effekte in der Schwangerschaft hin. Ältere Patienten und solche mit erhöhtem Risiko sollten JAKi nur nach sorgfältiger Nutzen‐Risiko‐Abwägung erhalten. Entsprechende Voruntersuchungen und regelmäßiges Labormonitoring sind notwendig, um eine sichere Therapie zu gewährleisten.

## WIRKMECHANISMUS VON JANUSKINASE‐INHIBITOREN (JAKi)

Chronisch entzündliche Erkrankungen werden molekularbiologisch durch unterschiedliche Entzündungskaskaden vermittelt. Proinflammatorische Zytokine wie Tumornekrosefaktor (TNF), Interleukin (IL)‐6 und IL‐1 initiieren die Produktion nachgeschalteter Mediatoren, darunter IL‐17A und IL‐22.^1^ Diese Botenstoffe binden an spezifische Rezeptoren auf der Zielzelle, wobei viele Zytokinrezeptoren den JAK‐STAT‐Signalweg zur intrazellulären Signalweiterleitung nutzen. Die Rezeptoreinheiten dimerisieren und initiieren so die intrazelluläre Signaltransduktion. Durch die Dimerisierung des Rezeptors werden Januskinasen (JAK) an dessen intrazelluläre Domänen rekrutiert und autophosphoryliert, was eine Konformationsänderung des Rezeptors zur Folge hat. Anschließend übertragen die aktivierten JAK Phosphatgruppen auf STAT‐Proteine (*signal transducers and activators of transcription*). Die nun ebenfalls phosphorylierten STAT werden dadurch aktiviert, dimerisieren und wandern in den Zellkern, um dort als Transkriptionsfaktoren Zielgene zu regulieren.

Über 50 unterschiedliche Zytokine und Wachstumsfaktoren vermitteln ihre Wirkung über den JAK/STAT‐Signalweg. Bei Menschen kommen vier unterschiedliche JAK vor: JAK1, JAK2, JAK3 und Tyrosinkinase (TYK) 2. Zytokine binden an ihre jeweiligen Rezeptoren, welche wiederum mit JAK‐ beziehungsweise TYK‐Molekülen assoziieren. Die jeweiligen Zytokin‐Rezeptor‐Komplexe verwenden entweder zwei JAK2‐Moleküle oder verschiedene Kombinationen aus JAK1, JAK2, JAK3 und TYK2. Dabei werden maximal drei verschiedene JAK aktiviert. Durch diese Aktivierung kommt es zur intrazellulären Signaltransduktion mit Transkription von Zielgenen durch STAT‐Dimere. So wirkt IL‐6 an den Zielzellen beispielsweise über seinen Rezeptorkomplex in Assoziation mit JAK1, JAK2 und TYK2. Je nach Rezeptorkomplex und JAK‐Cluster werden spezifische Signale übertragen und bestimmte Effekte in Zielzellen erzeugt (unter anderem Lymphozytenproliferation, Myelopoese oder Erythropoese).

Therapeutisch blockieren JAKi diese Signalkaskade: Die meisten JAKi binden kompetitiv an der ATP‐Bindungstasche der Kinase‐Domäne, sodass die Phosphorylierung sowie die weitere Signaltransduktion unterbunden werden. Januskinase‐Inhibitoren wirken daher, je nach Zieleinheit der JAK, immunmodulierend, antiproliferativ und entzündungshemmend.

Die erste Generation von JAK‐Inhibitoren (JAKi) war nicht hochselektiv für eine Januskinase: So hat Tofacitinib nicht nur eine hohe Affinität zu JAK3, sondern auch zu JAK1 und JAK2. Durch Weiterentwicklungen sind mittlerweile selektivere JAK‐Inhibitoren, zum Beispiel gegen JAK1 erhältlich, die eine spezifischere Hemmung des Zytokinrezeptorspektrums und ein im Vergleich zur ersten Generation von JAKi schmaleres Wirkungs‐ und Nebenwirkungsprofil erreichen.

Weniger selektive JAKi der ersten Generation, wie Tofacitinib, lassen sich von selektiveren Substanzen der zweiten Generation abgrenzen, etwa Upadacitinib, Abrocitinib und Filgotinib (JAK1‐Inhibitoren) oder Deucravacitinib (TYK2‐Inhibitor).^2^ Im Gegensatz zu anderen JAK‐Inhibitoren bindet Deucravacitinib nicht an die katalytische Kinase‐Domäne, sondern an die regulatorische Pseudokinase‐Domäne und hemmt dadurch selektiv TYK2. Durch die selektive Hemmung von TYK2 blockiert Deucravacitinib die Signalübertragung von IL‐12, IL‐23 und Typ‐I‐Interferonen; JAK1–3 bleiben in therapeutischen Dosierungen unbeeinträchtigt.^3^, ^4^

Über 50 Zytokine wirken an ihren Zielzellen nach Bindung ihrer jeweiligen Zytokin‐Rezeptor‐Komplexe über den intrazellulären JAK‐STAT‐Signalweg. Januskinase‐Inhibitoren (JAKi) blockieren diesen, indem sie die nachgeschaltete intrazelluläre Phosphorylierung von STAT‐Molekülen verhindern und diese ihre Funktion als Transkriptionsfaktoren verlieren. Neuere, selektivere JAKi wie Upadacitinib, Abrocitinib, Filgotinib und Deucravacitinib bieten gezieltere Wirkungen mit weniger Nebenwirkungen im Vergleich zur ersten Generation von JAKi.

## ANWENDUNGSGEBIETE VON JAKi

Aktuell sind systemische JAKi insbesondere im interdisziplinären Spektrum von chronisch entzündlichen beziehungsweise autoimmunen Erkrankungen zugelassen: Psoriasis, Psoriasis‐Arthritis (PsA), juvenile idiopathische Arthritis (JIA), Spondyloarthritis ankylosans (SpA), Colitis ulcerosa (CU), atopische Dermatitis (AD) und Alopecia areata (Tabelle 1).

**TABELLE 1:** Übersicht zu relevanten JAKi in der Dermatologie.^42^, ^43^, ^44^, ^45^, ^49^, ^50^, ^51^, ^52^, ^53^, ^55^, ^56^, ^57^

	Nichtselektiv innerhalb der JAK‐Familie					Selektiv innerhalb der JAK‐Familie				
Wirkstoff	Baricitinib	Tofacitinib	Ruxolitinib		Delgocitinib	Upadacitinib	Abrocitinib	Filgotinib	Deucravacitinib	Ritlecitinib
Fertigpräparat	Olumiant^®^	Xeljanz^®^	Jakavi^®^	Opzelura^®^ 15 mg/g	Anzupgo^®^ 20 mg/g	Rinvoq^®^	Cibinqo^®^	Jyseleca^®^	Sotyktu^®^	Litfulo^®^
Zugelassene Indikationen	AD, RA, AA	PsA, RA, SpA, JIA, CU	PV, Myelofibrose (Splenomegalie), GvHD	NSV	CHE, AD (USA, keine EU‐Zulassung, Stand 01/2025)	AD, PsA, RA SpA, CU, M. Crohn	AD	RA, CU	Pso	AA
Applikationsweg	Peroral	Peroral	Peroral	Kutan	Kutan	Peroral	Peroral	Peroral	Peroral	Peroral
Dosierung	AD: 4 mg 1 x/d 2 mg 1 x/d bei Risikopatienten oder gewünschter Dosisreduktion	PsA/RA/SpA: 5 mg 2 x/d	PV: 10 mg 2 x/d	NSV: läsional 2 x/d (max. 10% KOF pro Anwendung)	läsional 2 x/d (max. 5 g pro Anwendung)	AD, PsA: 15 mg 1 x/d Bei AD auch 30 mg 1 x/d je nach individuellem Krankheitsbild	AD: 200 mg 1 x/d 100 mg 1 x/d bei Risikopatienten oder gewünschter Dosisreduktion	RA: 200 mg 1 x/d	Pso: 6 mg 1 x/d	50 mg 1 x/d
Halbwertszeit	13 h	3 h	3 h	/	/	9–14 h	2–5 h	7 h	10 h	1,3–2,3 h
Affinität zu JAK‐Subtypen	JAK1, JAK2 > TYK2, JAK3	JAK1, JAK3 > JAK2	JAK1, JAK2		JAK1, JAK2, JAK3, TYK2	JAK1, JAK2	JAK1	JAK1	TYK2	JAK3, Kinasen der TEC‐Familie
Eliminierung	Ca. 70% renal	Ca. 70% hepatisch	Ca. 70% renal		Ca. 50/50% renal/hepatisch	Überwiegend hepatisch	Ca. 85% renal	Ca. 85% renal	Hepatisch > renal	Renal (ca. 66%) > hepatisch
Dosisanpassung bei *systemischer* Applikation und Nieren‐/Leberfunktionsstörung *Alle Präparate: Kontraindikation bei Leberfunktionsstörung Child Pugh C*	GFR 30–60 ml/min: 2 mg 1 x/d GFR < 30 ml/min: kontraindiziert	GFR < 30 ml/min und Hämodialyse: 5 mg 1 x/d Child Pugh B: 5 mg 1 x/d	GFR < 30 ml/min und Hämodialyse (nur an Dialysetagen nach Dialyse): 5 mg 1 x/d Child Pugh B: Dosishalbierung		/	Keine GFR‐Anpassung nötig.	GFR 30–60 ml/min: Dosishalbierung auf 100 bzw. 50 mg 1 x/d GFR < 30 ml/min: 50 mg 1 x/d	GFR 15–60 ml/min: 100 mg 1 x/d GFR < 15 ml/min: kontraindiziert	Keine GFR‐Anpassung nötig	Keine GFR‐Anpassung nötig

*Abk*.: AA, Alopecia areata; AD, atopische Dermatitis; CHE, chronisches Handekzem (hier mittelschwer bis schwer); CU, Colitis ulcerosa; GvHD, Graft versus Host Disease; KOF, Körperoberfläche; NSV, nicht segmentale Vitiligo; PV, Polycythaemia vera; PsA, Psoriasisarthritis; Pso, Psoriasis; RA, rheumatoide Arthritis; SpA, Spondylitis ankylosans; JIA, juvenile idiopathische Arthritis.

Bisher werden lediglich Ruxolitinib und Fedratinib als zugelassene JAKi auch zur Behandlung myeloproliferativer Erkrankungen eingesetzt. Ruxolitinib kann nach Zulassung durch die Europäische Arzneimittel‐Agentur (EMA) in der EU topisch zur Behandlung der nichtsegmentalen Vitiligo eingesetzt werden. In den USA ist das Präparat auch zur Therapie der AD ab 12 Jahren zugelassen. Nach Bestätigung der Sicherheit und Wirksamkeit in der 2024 publizierten Phase‐III‐Studie wurde der pan‐JAKi Delgocitinib zur topischen Therapie des moderaten bis schweren chronischen Handekzems durch die EMA zugelassen.^5^

Hinsichtlich der Pharmakokinetik werden JAKi nach oraler Applikation schnell und nahrungsunabhängig resorbiert: 30–60 Minuten nach oraler Verabreichung erreichen JAKi wie Tofacitinib oder Baricitinib ihre maximale Plasmakonzentration; die neueren JAKi binnen 4 Stunden.^2^ Je nach Präparat werden JAKi teils hepatisch, teils renal eliminiert. Eine entsprechende Prüfung von Medikamenteninteraktionen oder Anpassung an die aktuelle Nierenfunktion ist bei Verordnung daher notwendig. Januskinase‐Inhibitoren mit vorwiegend hepatischer Metabolisierung weisen eine kurze Halbwertszeit von circa 3 Stunden auf, so dass eine zweimalige Gabe pro Tag erforderlich ist. Januskinase‐Inhibitoren mit primär renaler Elimination weisen längere Halbwertszeiten auf, meist um 9–14 Stunden, sodass eine einmal tägliche Einnahme ausreichend ist.^6^

Aufgrund ihres raschen Wirkeintritts und ihrer kurzen Halbwertszeit – im Vergleich zu anderen systemischen Therapeutika – sind JAK‐Inhibitoren durch die tägliche orale Einnahme bei akuten Nebenwirkungen gut steuerbar. Prinzipiell eignen sie sich daher auch für eine intermittierende (off‐label) Therapie, etwa bei Patienten mit atopischer Dermatitis und saisonal begrenzten Beschwerden. Die orale Applikation und kurze Halbwertszeit bei Nebenwirkungen wie akuten Infektionen macht JAKi für Patienten sehr attraktiv.^7^ Zudem können mit Upadacitinib, Baricitinib und Abrocitinib jeweils, je nach klinisch individueller Ausprägung sowie Risikoprofil, zwei unterschiedliche Dosierungen angewendet werden.

Durch die gezielte Modulation des Immunsystems besitzen JAKi das Potenzial, das Management von verschiedenen komplexen entzündlichen und autoimmunen Erkrankungen deutlich zu verbessern.^8^ Dennoch können sie auch mit potenziellen Nebenwirkungen und Risiken verbunden sein, daher ist neben der Auswahl des passenden Patientenkollektivs und des geeigneten JAKi ein sorgfältiges Monitoring der Therapie unabdingbar.

Januskinase‐Inhibitoren werden zur Behandlung chronisch entzündlicher Erkrankungen wie Psoriasis, Psoriasisarthritis, Alopecia areata, chronischem Handekzem und atopischer Dermatitis eingesetzt; einige sind auch für myeloproliferative Erkrankungen zugelassen. Sie zeichnen sich durch eine schnelle Resorption und kurze Halbwertszeit aus, was eine flexible Dosierung und gute Steuerbarkeit bei Nebenwirkungen erlaubt. Trotz ihrer Wirksamkeit erfordert der Einsatz von JAKi ein sorgfältiges Monitoring aufgrund potenzieller Nebenwirkungen und Risiken.

## NICHTSPEZIFISCHE NEBENWIRKUNGEN

Trotz ihrer Wirksamkeit können JAK‐Inhibitoren unspezifische Nebenwirkungen hervorrufen, die für Patienten belastend sein können. Dazu zählen vor allem zu Beginn der Behandlung gastrointestinale Beschwerden wie Übelkeit, Erbrechen und Durchfall. Weitere mögliche Nebenwirkungen sind Kopfschmerzen, Schwindel und Müdigkeit, die meist bei weniger als 10 % der Patienten auftreten. Bei der topischen Anwendung können Juckreiz und Hautirritationen vorkommen.

Januskinase‐Inhibitoren können unspezifische Nebenwirkungen wie gastrointestinale Beschwerden, Kopfschmerzen, Schwindel und Müdigkeit verursachen, insbesondere zu Beginn der Behandlung. Hautirritationen und Juckreiz sind mögliche Nebenwirkungen bei topischer Anwendung.

## JAKi‐ASSOZIIERTE KUTANE NICHTINFEKTIÖSE NEBENWIRKUNGEN

**ABBILDUNG 1:**
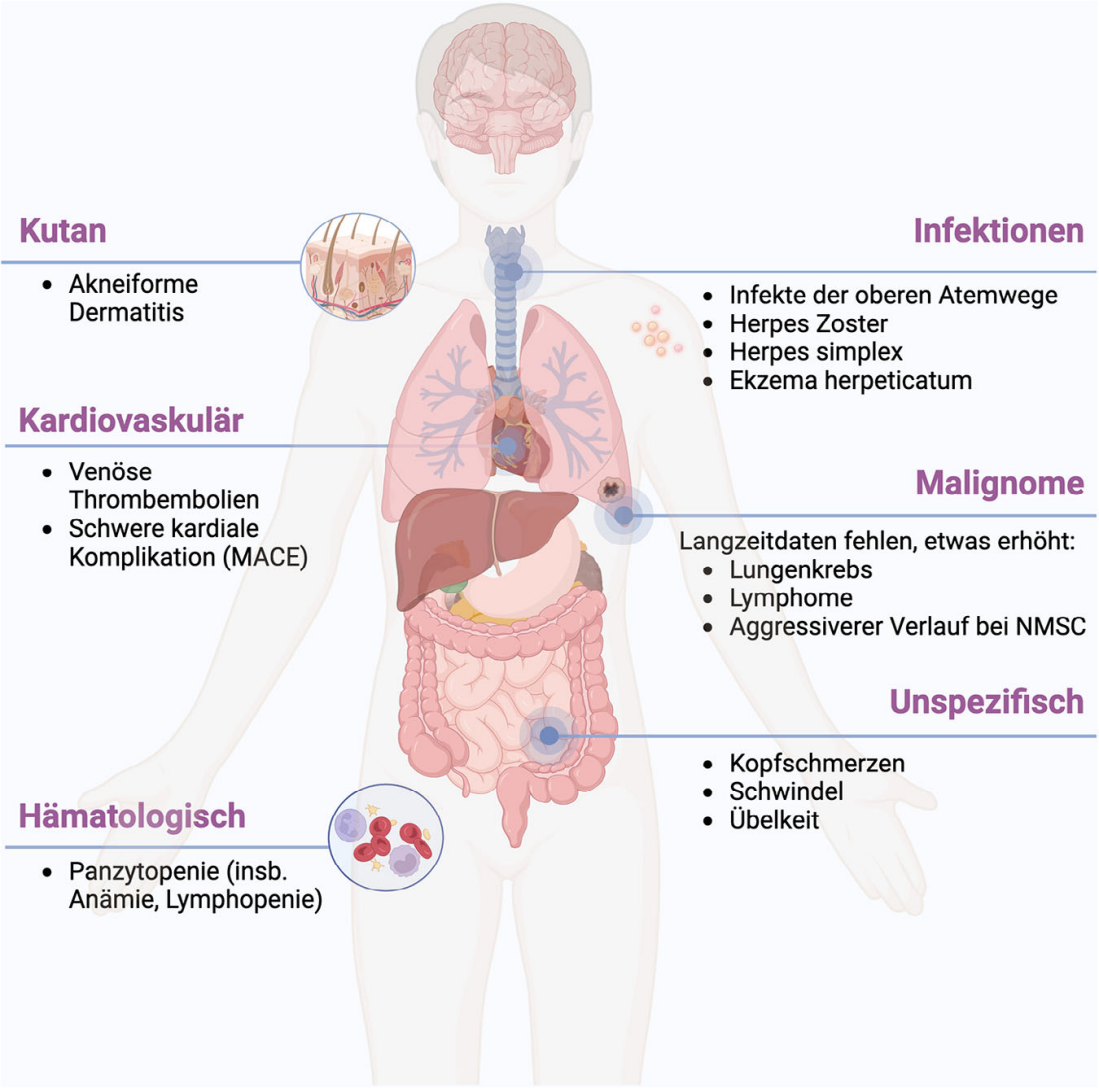
Mögliche Nebenwirkungen unter Januskinase‐Inhibitoren (Abbildung erstellt mit biorender.com). *Abk*.: MACE major adverse cardiac event; NMSC, non‐melanoma skin cancer

Sowohl die systemische als auch die topische Anwendung von JAKi sind mit vermehrtem Auftreten akneiformer Hautveränderungen assoziiert (Abbildung 1). In einer rezenten Metaanalyse mit über 10 000 eingeschlossenen Probanden berichteten 6,2% der Anwender von JAKi ein unerwünschtes Auftreten von Akne gegenüber 1,3% in der Kontrollgruppe.^9^ Bei Anwendern von Abrocitinib und Upadacitinib schienen höhere Dosen zudem häufiger akneiforme Läsionen hervorzurufen (< 2% bei 100 mg Abrocitinib vs. 4,7–5,8% bei 200 mg; 1,5% bei 15 mg Upadacitinib vs. 3,6% bei 30 mg).^10^ Bei der Mehrheit der Patienten sind Stirn und Wangen betroffen. In der Literatur wird – insbesondere bei Fehlen von Komedonen – die Einordnung der Hautveränderungen als der Rosazea ähnelnd diskutiert.^11^

Auch topische JAKi verursachten in bisherigen Studien zur AD oder Vitiligo in bis zu 6% der Fälle einen akneiformen Hautausschlag an den Auftragungsorten. Zudem berichteten knapp 6% der Anwender über lokal begrenzten Pruritus.

Die Empfehlungen zur Therapie der akneiformen Dermatitis orientiert sich an der Behandlung der Akne und Rosazea. In der Regel ist die topische Therapie mit Azelainsäure oder Benzoylperoxid ausreichend. Nur selten ist eine systemische Therapie der akneiformen Hautveränderungen mit Minocyclin oder Doxycyclin oder gar eine Unterbrechung der Therapie mit JAKi indiziert.^12^, ^13^

Die systemische und topische Anwendung von JAKi ist mit einem erhöhten Risiko akneiformer beziehungsweise der Rosazea ähnelnder Hautveränderungen verbunden. Deren Behandlung erfolgt in der Regel topisch mit Azelainsäure oder Benzoylperoxid, während eine systemische Behandlung oder Therapiepause der JAKi selten erforderlich ist.

## INFEKTIONSRISIKEN UNTER JAK‐INHIBITION

Zu den am häufigsten gemeldeten Infektionen bei etwa 5% der dermatologischen Patienten unter JAKi gehören Infektionen der oberen Atemwege. Die Inzidenzrate in verschiedenen klinischen Studien zu Abrocitinib und Upadacitinib bei Patienten mit AD lag bei 7%–9% beziehungsweise 6%–13%, verglichen mit 4%–5% beziehungsweise 4%–7% bei der Placebogruppe.^14^

Infekte der oberen Atemwege oder auch des Urogenitaltrakts sind jedoch unspezifisch und finden sich bei allen immunmodulatorischen Therapien. Bei Patienten mit AD, die Upadacitinib oder Abrocitinib erhielten, wurden häufiger Herpes‐simplex‐Virus (HSV)‐Infektionen beziehungsweise Reaktivierungen detektiert (2,1%–8,5% bei Upadacitinib vs. 0,4%–1,9% bei Placebo; 1%–2% bei Abrocitinib vs. 0% bei Placebo).^14^, ^15^, ^16^, ^17^

Eine Reaktivierung von VZV als Herpes‐zoster‐Infektion trat unter Upadacitinib im Nachbeobachtungszeitraum über 52 Wochen bei bis zu 5,6% der Patienten mit 30 mg täglich und bei bis zu 3,7% der Patienten mit täglicher Einnahme von 15 mg auf.^15^ Eine Herpes‐zoster‐Impfung, die in Deutschland ab dem 50. Lebensjahr bei bestimmten Grunderkrankungen erstattungsfähig ist, sollte daher vor Einleitung einer JAKi‐Therapie nachdrücklich empfohlen werden. Der Einfluss von JAKi auf die Signalwege von Interferonen und anderen antiviralen Zytokinen erklärt das vermehrte Auftreten von VZV‐Reaktivierungen.

Bei Patienten mit latenter Tuberkulose (TB) wurde über eine Reaktivierung bisher weder bei mit Abrocitinib noch bei mit Upadacitinib behandelten Patienten in den 12‐ beziehungsweise 16‐wöchigen Studienzeiträumen berichtet. Die Patienten müssen jedoch vor Therapieeinleitung ein TB‐Screening erhalten (*Interferon‐Gamma‐Release Assay*, IGRA). Bei einer aktiven TB darf verständlicherweise keine Therapie mit JAKi erfolgen. Bei einer zuvor unbehandelten, latenten TB sollte vor Einleitung der Therapie eine Chemoprävention begonnen werden (Tabelle 2). Die Röntgenuntersuchung des Thorax wird laut aktueller Fachinformationen nicht im Standard‐Screening gefordert, hilft jedoch zur weiteren diagnostischen Abklärung bei positivem IGRA.

**TABELLE 2:** Monitoring verschiedener JAKi in häufiger dermatologischer Anwendung entsprechend der Fachinformationen.^42^, ^43^, ^44^, ^45^, ^55^, ^56^, ^58^

Wirkstoff	Baricitinib	Tofacitinib	Upadacitinib	Abrocitinib	Ritlecitinib	Deucravacitinib	Ruxolitinib	Delgocitinib
Fertigpräparat	Olumiant^®^	Xeljanz^®^	Rinvoq^®^	Cibinqo^®^	Litfulo^®^	Sotyktu^®^	Opzelura^®^ 15 mg/g topisch	Anzupgo^®^ 20 mg/g topisch
Screening vor Therapiebeginn	Diff. BB, Transaminasen, Impfstatus, TB‐Screening, Screening virale Hepatitis, Erwägung Impfung gegen Herpes Zoster					TB‐Screening, Impfstatus, Transaminasen (nicht bei schwerer Leberfunktionsstörung)	Gilt nicht für Ruxolitinib topisch	Nicht erforderlich
Konsequenz Screening‐Befunde	Bei aktiver und latenter TB Behandlung initiieren, Impfstatus aktualisieren, kein Therapiestart bei aktiver Virushepatitis^*^						Gilt nicht für Ruxolitinib topisch	
	Kein Therapiestart bei Anämie (< 8 g/dl), Lymphopenie (< 500/mm^3^), Neutropenie (< 1000/mm^3^)	Kein Therapiestart bei Anämie (< 9 g/dl bei Erwachsenen, < 10 g/dl bei Kindern), Lymphopenie (< 750/mm^3^), Neutropenie (< 1000 Zellen/mm^3^, < 1200 Zellen/mm^3^ bei Kindern)	Kein Therapiestart bei Anämie (< 8 g/dl), Lymphopenie (< 500/mm^3^), Neutropenie (< 1000/mm^3^)	Kein Therapiestart bei Anämie (< 10 g/dl), Thrombopenie (150 000/mm^3^), Lymphopenie (< 500/mm^3^), Neutropenie (< 1200/mm^3^)	Kein Therapiestart bei Thrombopenie (100 000/mm^3^), Lymphopenie (< 500/mm^3^)			
**Empfehlung Monitoring**:								
Diff. BB	Im Rahmen der Routineuntersuchung	Nach 4–8 Wochen, dann alle 3 Monate	Im Rahmen der Routineuntersuchung	4 Wochen nach Therapiestart, dann im Rahmen der Routineuntersuchung		Keine Empfehlungen	Keine Empfehlungen	Nicht erforderlich
Serumlipide	12 Wochen nach Therapiestart, dann nach Leitlinie Hyperlipidämie	8 Wochen nach Therapiestart, dann nach Leitlinie Hyperlipidämie	12 Wochen nach Therapiestart, dann nach Leitlinie Hyperlipidämie	4 Wochen nach Therapiestart, dann nach Leitlinie Hyperlipidämie	Keine Empfehlungen			
Transaminasen	Im Rahmen der Routineuntersuchung		Im Rahmen der Routineuntersuchung		Keine Empfehlungen			
Indikationen für Therapiepause	Schwere Infektion						Gilt nicht für Ruxolitinib topisch	Ausstehende Besserung nach 12‐wöchiger Therapie
	Arzneimittelbedingte Leberschädigung^**^ Anämie (< 8 g/dl) Lymphopenie (< 500/mm^3^), Tofacitinib (Dosisreduktion/Pause bei 500–1000/mm^3^, Therapieende bei zweimalig < 500/mm^3^l) Neutropenie (< 1000/mm^3^), Tofacitinib (Dosisreduktion/Pause bei <750/mm^3^, Therapieende bei zweimalig < 500/mm^3^) Thrombopenie (50 000/ mm^3^)^***^				Thrombopenie (100 000/mm^3^) Lymphopenie (< 500/mm^3^)			

*Abk*.: Diff. BB, Differentialblutbild; TB, Tuberkulose

* In Fachinformation Deucravacitinib nicht explizit erwähnt (Vorsicht bei chronischen Infektionen).

** In Fachinformation Abrocitinib nicht speziell erwähnt.

*** Nur in Fachinformation Abrocitinib erwähnt.

Das Risiko einer schweren Infektion, die beispielsweise zur Hospitalisation führt, wird häufig als das wichtigste Sicherheitsergebnis bei Studien mit Immuntherapeutika angesehen. Hierzu gehören etwa Pneumonien, Herpangina und Eczema herpeticatum. Die absoluten Ereignisraten für schwere Infektionen waren niedrig (zwei bis vier Ereignisse pro 100 Personenjahre Nachbeobachtungszeit) in den JAK1‐Studien und entsprechen in etwa denen von TNF‐Inhibitoren. Ausnahmen hiervon sind jedoch Reaktivierungen von latenten Viren, wie VZV, HSV und Cytomegalievirus.^14^

Bei der Behandlung der AD mit Upadacitinib scheint das Risiko schwerer Infektionen dosisabhängig zu sein: Für die tägliche Einnahme von 30 mg wurden 3–4 Ereignisse pro 100 Personenjahre berichtet, gegenüber 2–3 Ereignissen bei der 15‑mg‐Dosierung. Hinsichtlich opportunistischer Infektionen beschreiben Winthrop et al. im Indikationsgebiet der rheumatoiden Arthritis (RA) ebenfalls ein geringes, aber dosisabhängig vermehrtes Aufkommen. Systematische Analysen mit Langzeitdaten zu deren Auftreten bei dermatologischen Indikationen fehlen derzeit noch.^18^

Unter der Therapie mit JAKi besteht ein erhöhtes Risiko für Infektionen, insbesondere der oberen Atemwege und Reaktivierungen von Herpesviren wie HSV und VZV. Eine Herpes‐Zoster‐Impfung wird vor Therapiebeginn empfohlen und Patienten müssen auf latente Tuberkulose gescreent werden. Das Risiko schwerer Infektionen, einschließlich Pneumonien, entspricht etwa dem unter TNF‐Inhibitoren und ist je nach JAKi teils dosisabhängig.

## LIPIDPROFIL UND KARDIOVASKULÄRES RISIKO

Hinsichtlich des Lipidprofils können die systemischen JAKi Abrocitinib, Baricitinib, Tofacitinib und Filgotinib in den empfohlenen Dosierungen zu einem Anstieg der HDL‐ und LDL‐Werte führen. Dies betrifft bis zu vier von zehn Patienten und erfordert entsprechendes leitliniengerechtes Management der Hyperlipidämie.^10^, ^19^ Ob hierdurch ein langfristig erhöhtes kardiovaskuläres Risiko besteht, ist aufgrund der fehlenden Datenlage noch unklar.

In der *ORAL‐Surveillance‐Studie*, einer der ersten Sicherheitsstudien zu JAK‐Inhibitoren, wurde für Tofacitinib (2 x 5 mg bzw. 2 x 10 mg täglich) im Vergleich zu TNF‐Inhibitoren ein erhöhtes Risiko für thromboembolische Ereignisse beschrieben.^14^ Die zugrunde liegende molekulare Pathophysiologie ist bislang unklar. Zu beachten ist, dass in der ORAL‐Surveillance‐Studie Patienten mit bereits bestehendem kardiovaskulärem Risiko eingeschlossen wurden.

Weiterführende Beobachtungsstudien und Analysen konnten bisher das bei rheumatologischen Indikationen beobachtete erhöhte kardiovaskuläre Risiko von JAKi im dermatologischen Kontext nicht bestätigen, sodass die Datenlage hierzu aktuell unzureichend bleibt.^14^

In einer systematischen Übersichtsarbeit und Metaanalyse von 35 randomisierten klinischen Studien mit über 20 000 Patienten mit dermatologischen Erkrankungen fanden Ingrassia et al. kein erhöhtes Risiko für schwerwiegende unerwünschte kardiale Ereignisse (*major adverse cardiac events*, MACE), venöse Thromboembolien (VTE) oder die Gesamtmortalität unter JAK‐Inhibitoren im Vergleich zu Placebo oder einem aktiven Vergleichsmedikament.^9^ Auch unter JAKi‐Anwendern mit AD wurden gegenüber Vergleichspräparaten nur selten Fälle von MACE berichtet.^15^ Eine weitere Übersichtsarbeit mit über 450 000 Patienten aus Kohorten und randomisierten klinischen Studien ergab weder eine signifikante Assoziation von AD mit dem Auftreten von VTE noch ein erhöhtes Risiko für das Auftreten von VTE bei Teilnehmern mit AD, die JAKi erhielten.^20^

Eine weitere Übersichtsarbeit zum Sicherheitsprofil von Upadacitinib bei rheumatologischen Indikationen (RA, PsA, SpA) berichtete vergleichbare Raten für MACE und VTE wie unter den Vergleichstherapien mit Adalimumab und Methotrexat.^15^ Dort beschriebene potenzielle Risikofaktoren für MACE und VTE bei dieser Kohorte unter JAKi sind bereits bekannte Risikofaktoren für kardiovaskuläre Ereignisse wie arterielle Hypertonie, Diabetes mellitus oder Nikotinabusus. Zudem besteht eine höhere Inzidenz von VTE bei rheumatologischen Indikationen unter JAKi als bei dermatologischen Indikationen.^20^, ^21^

Systemische JAKi können zu erhöhten HDL‐ und LDL‐Werten führen, was ein leitliniengerechtes Management der Hyperlipidämie erfordert. Obwohl ein erhöhtes Risiko für Thrombembolien unter Tofacitinib bei RA beschrieben wurde, konnte ein erhöhtes kardiovaskuläre Risiko von anderen JAKi bei sowohl rheumatologischen und dermatologischen Indikationen nicht eindeutig bestätigt werden. Beobachtungsstudien und Metaanalysen zeigen bisher kein signifikant erhöhtes Risiko für MACE oder VTE im Vergleich zu Placebo oder Vergleichsmedikamenten.

## HÄMATOLOGISCHE VERÄNDERUNGEN

Insbesondere JAK2 ist an pleiotropen Signalkaskaden beteiligt, die wesentlich zur Aufrechterhaltung einer gesunden Hämatopoese beitragen. Dies zeigt sich eindrücklich in Tierversuchen: JAK2‐defiziente Mäuse sterben bereits in utero infolge eines Knochenmarkversagens.^22^ Januskinase 2 wird durch verschiedene Zytokine aktiviert, die unterschiedliche hämatopoetische Prozesse im Knochenmark steuern – darunter die Granulopoese (Granulozyten‐Kolonie‐stimulierender Faktor, IL‐3, Granulozyten‐Makrophagen‐Kolonie‐stimulierender Faktor), Erythropoese (Erythropoietin), Thrombopoese (Thrombopoietin) und Eosinopoese (IL‐5).^23^ Im pathologischen Kontext von myeloproliferativen Erkrankungen, wie Polycythaemia vera, liegt oft eine Gain‐of‐Function‐Mutation im *JAK2*‐Gen zugrunde.^24^

So wird für die Behandlung von *JAK2*‐mutierten Patienten mit myeloproliferativen Erkrankungen die medikamentöse Hemmung von JAK2 mit Ruxolitinib, Fedratinib und Momelotinib genutzt. Bei anderen Patientenkollektiven ohne *JAK2*‐Mutation kann jedoch eine Zytopenie als dosisabhängige Nebenwirkung von JAKi der ersten Generation auftreten.^25^, ^26^, ^27^

Januskinase‐Inhibitoren der zweiten Generation mit zunehmender Selektivität für JAK1, wie Upadacitinib oder Filgotinib, umgehen dieses Nebenwirkungsprofil weitgehend.^28^ Auch bei der Behandlung mit dem TYK2‐Inhibitor Deucravacitinib sind Zytopenien bei fehlender Inhibition von JAK2 nicht als Komplikation beobachtet worden. Ein Labormonitoring ist daher nicht bei allen JAKi vorgeschrieben. Dennoch ist die Untersuchung des Differenzialblutbilds vor Therapieeinleitung und wenn medizinisch erforderlich in Intervallen sinnvoll. Bei Auftreten einer Anämie oder eines Hämoglobinabfalls von > 2 g/dl, einer Lymphopenie oder Neutropenie sollte die Medikation entsprechend der jeweiligen Fachinformation pausiert werden (Tabelle 2).^27^

Januskinasen, insbesondere JAK2, spielen eine zentrale Rolle in der Hämatopoese, und ihre Hemmung kann zu dosisabhängigen Zytopenien führen, besonders bei JAKi der ersten Generation. Selektivere JAKi der zweiten Generation sowie der TYK2‐Inhibitor Deucravacitinib zeigen ein geringeres Risiko für hämatologische Nebenwirkungen. Ein Differenzialblutbild sollte vor Therapieeinleitung und je nach Empfehlung der Fachinformation oder klinischen Notwendigkeit während der Behandlung überwacht werden.

## EINFLUSS AUF MALIGNOME

Eine langfristige Suppression des JAK/STAT‐Signalwegs mag aus mechanistischer Perspektive das Risiko für Malignome erhöhen, da Typ‐I‐ und Typ‐II‐Interferone eine wichtige Rolle in der antitumoralen Kontrolle des Immunsystems spielen. Auf der anderen Seite ist der JAK/STAT‐Signalweg auch bei Zellüberleben und Proliferation von Bedeutung, so dass die Konsequenzen in Tumoren unterschiedlich ausfallen können. Hier sind weitere Untersuchungen und langjährige Beobachtungsdaten erforderlich.

Besonders auffällig war eine erhöhte Inzidenz von Bronchialkarzinomen und Lymphomen in der Tofacitinib‐Gruppe im Vergleich zur mit TNF‐Inhibitoren behandelten RA‐Kontrollgruppe (*Hazard Ratio* 1,48). In diesem Zusammenhang sollte bedacht werden, dass bei Patienten mit RA eine erhöhte Inzidenz von Hodgkin‐ und Non‐Hodgkin‐Lymphomen im Vergleich zur Normalbevölkerung bekannt ist.^29^ Eine stratifizierte *Post‐hoc*‐Analyse zeigte zudem, dass das vermehrte Auftreten von Malignomen unter Tofacitinib im Vergleich zu TNF‐Inhibitoren ausschließlich in der Hochrisikogruppe beobachtet wurde (Alter über 65 Jahre, aktive oder ehemalige Langzeitraucher), nicht jedoch in der Niedrigrisikogruppe.^30^ In der Analyse wurde nichtmelanozytärer Hautkrebs (NMSC) nicht untersucht.^31^

Patienten mit Organtransplantation, die mit Tofacitinib behandelt wurden, wiesen ein höheres Risiko für lymphoproliferative Malignome auf. Dabei ist zu berücksichtigen, dass in den Studien höhere Tofacitinib‐Dosen verwendet wurden als die von der EMA für die Behandlung der rheumatoiden Arthritis zugelassene Dosis und dass die Patienten zusätzlich mit weiteren Immunsuppressiva behandelt wurden.^27^

Die Raten melanozytärer als auch nichtmelanozytärer Hautkrebse scheinen unter JAKi erhöht zu sein: In den 5‐Jahres‐Beobachtungszeiträumen traten bei Patienten mit RA unter Upadacitinib fünfmal so viele NMSC wie unter Adalimumab auf (0,5 vs. 0,1 Ereignisse pro 100 Patientenjahre).^32^, ^33^ Eine Analyse der Arzneimittelsicherheitsdatenbank der Weltgesundheitsorganisation meldete ein positives Disproportionalitätssignal – also einen Verdacht auf einen Zusammenhang zwischen Arzneimittel und unerwünschtem Ereignis – für verschiedene maligne Hauttumoren unter JAKi. Bei Plattenepithelkarzinomen wurden entsprechende Signale für Ruxolitinib (IC_025_ = 3,92) und Tofacitinib (IC_025_ = 0,82) registriert. Für Melanome ergaben sich Signale bei Ruxolitinib (IC_025_ = 0,81) und Tofacitinib (IC_025_ = 0,74). Beim Merkelzellkarzinom zeigten sich positive Disproportionalitätssignale unter Ruxolitinib (IC_025_ = 4,00), Tofacitinib (IC_025_ = 1,01) und Baricitinib (IC_025_ = 0,53). Darüber hinaus war das Merkelzellkarzinom als grundsätzlich seltene Erkrankung in der gemeldeten Stichprobe besonders stark vertreten und wurde bei allen untersuchten JAKi mit einem signifikanten Disproportionalitätssignal in Verbindung gebracht.^34^, ^35^ Eine weitere epidemiologische Studie basierend auf dem *US Food and Drug Administration Adverse Event Reporting System* (FAERS) bestätigte eine positive Assoziation von Ruxolitinib, Tofacitinib und Upadacitinib mit bösartigen Hauttumoren. Im Speziellen wurde eine signifikante Assoziation von neuroendokrinen Hauttumoren mit Ruxolitinib sowie Plattenepithelkarzinom der Haut mit Upadacitinib berichtet.^36^ Ein mögliches Risiko von JAKi für die Photokarzinogenese der Haut muss noch geklärt werden.

Auch wenn bisher keine systematische Auswertung vorliegt, sind neben dem gehäuften Auftreten von NMSC unter JAKi vermehrt Fälle besonders aggressiv wachsender epithelialer Tumoren beschrieben worden – insbesondere kutane Plattenepithelkarzinome unter oraler Behandlung mit Ruxolitinib.^37^ Allerdings reichen die bislang verfügbaren Daten für eine eindeutige Assoziation nicht aus, was unter anderem auf das Fehlen von Registerstudien zu NMSC zurückzuführen ist.^38^

Eine langfristige Hemmung des JAK/STAT‐Signalwegs könnte das Risiko für Malignome erhöhen, insbesondere in Hochrisikogruppen wie älteren oder langzeitrauchenden Patienten. Bei Patienten mit RA unter Tofacitinib zeigte sich im Vergleich zu TNF‐Inhibitoren ein erhöhtes Risiko für Lungenkrebs und Lymphome. Beobachtungsstudien berichten von gehäuftem Auftreten von bösartigen Hauttumoren unter JAKi.

## SCHWANGERSCHAFT, STILLZEIT, FERTILITÄT

Auf Basis von Tierversuchen bergen JAKi in der Schwangerschaft teratogene Effekte. Genetisch manipulierte Mäuse mit funktionellen JAK1‐ oder JAK2‐Defizienzen sind nicht überlebensfähig. Auch sind beim Menschen keine kompletten Loss‐of‐Function‐Mutationen von JAK1 oder JAK2 bisher beschrieben.^39^

Es wird angenommen, dass JAKi die Plazentaschranke bereits zu Beginn der Schwangerschaft passieren können. In Tierstudien zeigte Tofacitinib teratogene und fetozide Wirkungen, wenn es in Dosierungen oberhalb der für den Menschen zugelassenen Menge verabreicht wurde. Experimentell wurde Tofacitinib in der Milch säugender Ratten nachgewiesen.^40^ Umfangreichere Humanstudien zur Sicherheit von JAKi während der Schwangerschaft oder Stillzeit fehlen bisher, sodass ihre Anwendung bei diesen Patientinnen unbedingt vermieden werden sollte.^27^ Frauen im gebärfähigen Alter müssen aufgrund der aktuellen Datenlage aufgefordert werden, eine zuverlässige Kontrazeption durchzuführen, wenn die Einnahme von JAKi geplant ist. Auch bis 1–4 Wochen nach der letzten Dosis ist eine zuverlässige Verhütungsmethode anzuwenden.

Bezüglich der männlichen Fertilität wurden unter Filgotinib verringerte Fertilität, eingeschränkte Spermatogenese und histopathologische Veränderungen männlicher Fortpflanzungsorgane beobachtet, die dosisabhängig irreversibel waren. Zwei randomisierte kontrollierte Humanstudien (MANTA und MANTA‐RAY) deuten jedoch darauf hin, dass die tägliche Gabe von 200 mg Filgotinib über einen Zeitraum von 13 Wochen keinen messbaren Einfluss auf Spermaparameter oder Sexualhormone bei Männern mit aktiver chronisch entzündlicher Darmerkrankung oder entzündlich‐rheumatischen Erkrankungen hat.^41^ Das potenzielle Risiko einer verringerten Fertilität oder Infertilität unter Filgotinib sollte daher vor Behandlungsbeginn mit männlichen Patienten besprochen werden.

Abrocitinib, Baricitinib, Upadacitinib und Tofacitinib zeigten im Tiermodell keinen Einfluss auf die männliche Fertilität. Der Einfluss auf die Fertilität beim Menschen wurde nicht untersucht.^42^, ^43^, ^44^, ^45^

JAKi bergen laut Tierstudien in der Schwangerschaft teratogene Effekte und sollten daher vermieden werden. Frauen im gebärfähigen Alter sollten während und nach der Behandlung mit JAKi eine zuverlässige Verhütung anwenden. Ein negativer Einfluss von Filgotinib auf die männliche Fertilität wird derzeit diskutiert, doch Humanstudien zeigen keinen signifikanten Effekt auf Spermaparameter bei empfohlener Dosierung; andere JAKi beeinflussten die männliche Fertilität im Tiermodell nicht.

## AKTUELLE EMPFEHLUNGEN

Ein interdisziplinäres Expertengremium aus Ärzten und Patientenvertretern überprüfte kürzlich die Daten der ORAL‐Surveillance‐Studie zu Tofacitinib sowie Beobachtungsdaten zu Baricitinib. Auf Basis der vorliegenden Daten kamen die Experten zu dem Schluss, dass die beschriebenen Risiken für alle Januskinase‐Inhibitoren mit zugelassener Indikation bei chronisch‐entzündlichen Erkrankungen gelten. Auf Basis dieser Übersicht sprach das *Pharmacovigilance Risk Assessment Committee* (PRAC) der EMA spezielle Empfehlungen zur Minimierung des Risikos schwerer Nebenwirkungen von JAKi bei chronisch‐entzündlichen Erkrankungen aus.^46^ Zum Zeitpunkt der veröffentlichten PRAC‐Empfehlungen war der TYK2‐Inhibitor Deucravacitinib noch nicht zugelassen und daher nicht Teil des Bewertungsverfahrens. Im klinischen Alltag sollte sein Sicherheitsprofil dennoch berücksichtigt werden – auch wenn es sich als Pseudokinase‐Inhibitor von den bisherigen JAKi durch ein anderes Risikoprofil unterscheidet.^46^

Aufgrund der gelisteten Nebenwirkungen sollen JAKi bei gefährdeten Patientengruppen nur dann angewendet werden, wenn eine Behandlungsalternative fehlt. Zum diesem Klientel gehören Patienten im Alter von ≥ 65 Jahren, Patienten mit erhöhtem Risiko für schwere Herz‐Kreislauf‐Probleme (zum Beispiel Herzinfarkt oder Schlaganfall), Patienten, die rauchen oder in der Vergangenheit lange geraucht haben sowie Patienten mit erhöhtem Risiko für Krebs oder VTE. Erwähnenswert ist, dass in der ORAL‐Surveillance‐Studie zu Tofacitinib – die als Grundlage für die genannten Empfehlungen diente – ein kardiovaskulärer Risikofaktor als Einschlusskriterium erforderlich war. Besonders im Studienarm mit einer Dosierung von 2 x 10 mg täglich wurde ein erhöhtes Risiko für kardiovaskuläre Ereignisse festgestellt.^14^, ^47^

Patienten, die nicht zur oben genannten Hochrisikogruppe gehören, aber Risikofaktoren für venöse Thromboembolien, Malignome oder schwere kardiovaskuläre Ereignisse aufweisen, sollten nach Möglichkeit eine reduzierte Dosis erhalten.

**ABBILDUNG 2:**
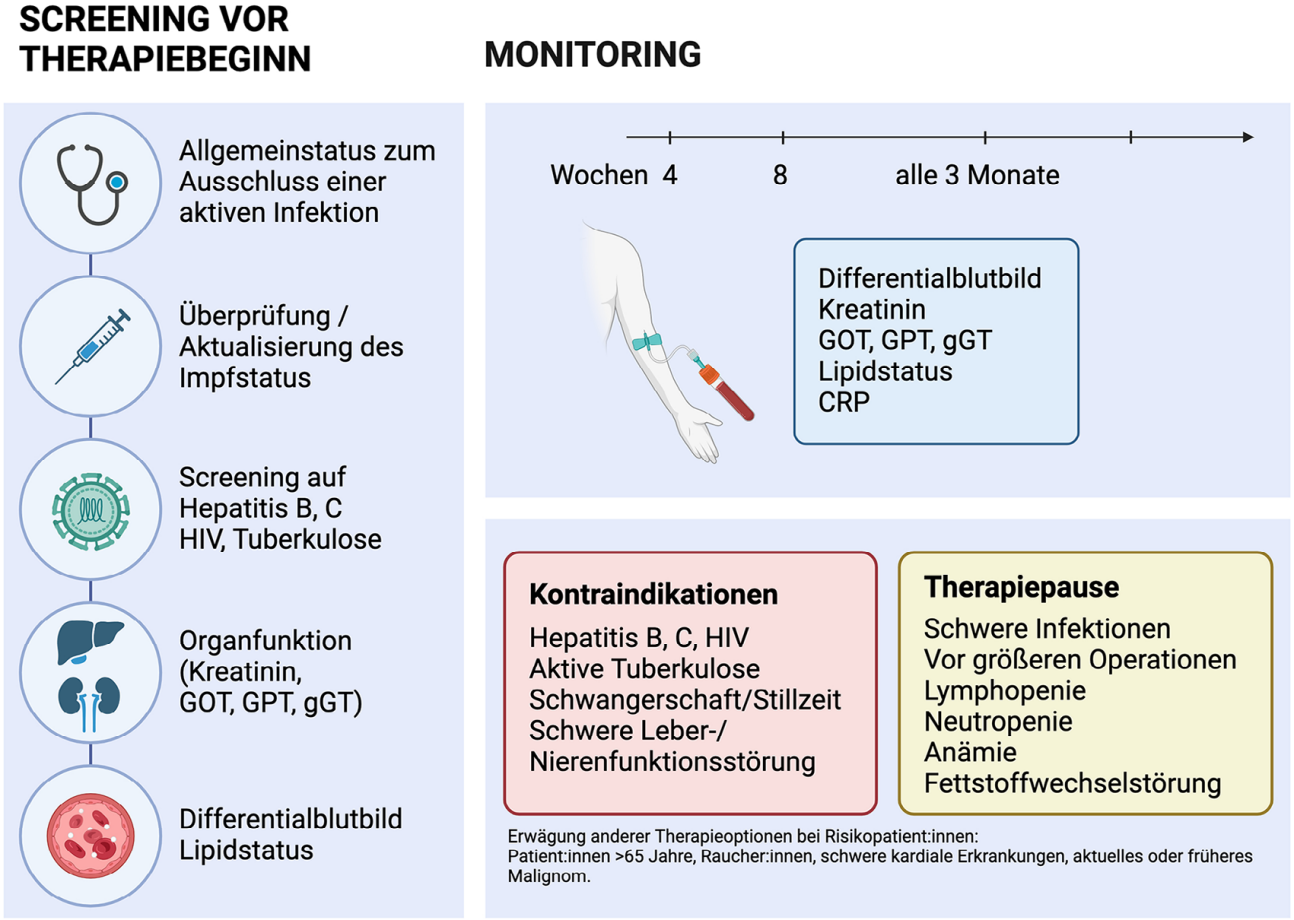
Orientierender Überblick über Therapievorbereitung und Monitoring unter JAKi (Abbildung erstellt mit biorender.com).^42^, ^43^, ^44^, ^45^, ^49^, ^50^, ^51^, ^52^, ^53^

Zudem sollen Patienten, die JAKi einnehmen, über das Risiko von Hauttumoren aufgeklärt werden und regelmäßig ein Hautkrebsscreening erhalten. Insbesondere bei Plattenepithelkarzinomen mit hohem Risiko in der Vorgeschichte oder bei zusätzlichen Risikofaktoren wie hämatoonkologischen Grunderkrankungen sind aggressive Tumorverläufe möglich. Eine orientierende Übersicht zur Therapievorbereitung und zum Screening unter JAKi ist in Abbildung 2 dargestellt. Für Deucravacitinib liegen keine Herstellerempfehlungen bezüglich eines Labormonitorings unter Therapie vor. Für Tofacitinib, Baricitinib, Ruxolitinib, Upadacitinib und Abrocitinib sind in Tabelle 2 die Empfehlungen zum Screening vor Therapiebeginn sowie zum Monitoring während der Behandlung und in Tabelle 3 die Kontraindikationen und besonderen Warnhinweise gemäß den jeweiligen Fachinformationen zusammengefasst. Zur Risikominimierung beim Einsatz von JAKi bei dermatologischen Patienten erarbeiteten Wohlrab et al. interdisziplinär eine Checkliste, welche im klinischen Alltag der pragmatischen und dennoch sorgfältigen Identifikation von Risikopatienten dient.^48^

Januskinase‐Inhibitoren sollten bei Risikopatienten – etwa älteren Personen, Rauchern sowie Patienten mit kardiovaskulären oder onkologischen Risikofaktoren – nur dann eingesetzt werden, wenn keine geeigneten Therapiealternativen zur Verfügung stehen. Bei Patienten mit Risikofaktoren für Thromboembolien, Malignome oder kardiovaskuläre Ereignisse wird eine reduzierte Dosierung empfohlen. Zudem sollten Patienten über das erhöhte Risiko für Hauttumoren aufgeklärt und regelmäßig daraufhin gescreent werden. Detaillierte Empfehlungen zum Screening und Therapiemonitoring sind in der jeweiligen Fachinformation des Präparats enthalten.

**TABELLE 3:** Kontraindikationen und spezielle Warnhinweise verschiedener JAKi in häufiger dermatologischer Anwendung entsprechend der Fachinformationen.^42^, ^43^, ^44^, ^45^, ^53^, ^57^

Wirkstoff	Baricitinib	Tofacitinib	Upadacitinib	Abrocitinib	Ritlecitinib	Deucravacitinib	Ruxolitinib	Delgocitinib
Fertigpräparat	Olumiant^®^	Xeljanz^®^	Rinvoq^®^	Cibinqo^®^	Litfulo^®^	Sotyktu^®^	Opzelura^®^ 15 mg/g topisch	Anzupgo^®^ 20 mg/g topisch
Absolute Kontraindikationen	Schwangerschaft/Stillzeit Überempfindlichkeit gegen Wirkstoff/Bestandteile							Überempfindlichkeit gegen Wirkstoff/ Bestandteile
	Aktive TB/schwerwiegende Infektionen Schwere Leberfunktionsstörung					Bei Patienten mit schwerer Leberfunktionsstörung nicht empfohlen	Gilt nicht für Ruxolitinib	
Besondere Warnhinweise	Bei unbehandelter latenter TB, Therapie initiieren Keine Lebendimpfungen unter Therapie Risikofaktoren für Malignome, nur wenn keine Therapiealternativen^*^ Anwendung mit Vorsicht bei Patienten ≥ 65 Jahren^**^ Anwendung nur nach Nutzen‐Risiko‐Abwägung: Atherosklerose, kardiovaskuläre Risikofaktoren (Raucher/ehemalige Langzeitraucher)^**^ Anwendung nur nach Nutzen‐Risiko‐Abwägung: bei chronischen/rezidivierenden Infektionen/ Exposition gegenüber TB^***^ Herkunft aus/Reisen in Gebiete mit endemischer TB/Mykosen^*^, ^***^				Bei unbehandelter latenter TB, Therapie initiieren Vermeidung von Lebendimpfungen unter Therapie Vorsicht bei Patienten mit erhöhtem Risiko für Thromboembolien	Bei latenter TB, Therapie initiieren Keine Lebendimpfungen unter Therapie Anwendung mit Vorsicht bei Patienten ≥75 Jahren Vorsicht bei Patienten mit chronischen Infektionen	Nicht zur Anwendung am Auge/oralen Einnahme/intravaginalen Anwendung	Nicht zur Anwendung an Auge, Mund und anderen Schleimhäuten Zur Vorsicht keine Anwendung in der Schwangerschaft Bei Anwendung in Stillzeit nach Auftragen der Creme Kontakt mit Brustwarze vermeiden
Vorsichtsmaßnahmen während Therapie	Kontrazeption bei Frauen im gebärfähigen Alter während Therapie und bis 4 Wochen nach Therapieende Regelmäßige Hautkrebsvorsorge Abrocitinib: Kombination mit starken Immunsuppressiva meiden/nicht untersucht					Keine Empfehlungen	Kontrazeption Hautkrebsvorsorge	Regelmäßige Untersuchungen der Applikationsstelle
	/	Beachtung von Wechselwirkungen CYP3A4	Beachtung von Wechselwirkungen CYP3A4	Beachtung von Wechselwirkungen CYP2C9/CYP2C19	Beachtung von Wechselwirkungen CYP3A4, CYP1A2			

*Abk*.: TB, Tuberkulose

* In Fachinformationen Deucravacitinib nicht erwähnt/mangelnde Datenlage.

** Gilt nicht für Baricitinib.

*** Für Tofacitinib und Baricitinib nicht speziell in Fachinformation erwähnt.

## FAZIT

Januskinase‐Inhibitoren sind *small molecules*, die aktuell bei einigen entzündlichen dermatologischen Krankheitsbildern sehr erfolgreich eingesetzt werden. Die Zahl der dermatologischen Indikationen und der behandelten Patienten wird in Zukunft deutlich zunehmen.^54^

Aufgrund des nicht zu vernachlässigenden Sicherheitsprofils sind transparente Aufklärung der Patienten, gründliche Therapievorbereitung als auch adäquates Monitoring von Nebenwirkungen im Therapieverlauf unerlässlich. Auch die Abfrage der bereits jetzt empfohlenen Kriterien und Impfungen sollte schriftlich dokumentiert werden. Generell sollte auch der Impfstatus – nicht nur von VZV – vor Therapieeinleitung geprüft und aktualisiert werden. Die Evaluation der Langzeitsicherheit wird erst nach umfangreichen Langzeitstudien möglich sein. Mögliche Risiken bei Langzeitbehandlungen von Erwachsenen und Kindern mit JAKi sind derzeit nicht sichtbar.

## DANKSAGUNG

Open access Veröffentlichung ermöglicht und organisiert durch Projekt DEAL.

## INTERESSENKONFLIKT

A.R.K. gibt an, in den letzten 5 Jahren Honorare für Vorträge von Recordati Rare Diseases Germany erhalten zu haben. J.H. gibt an, in den letzten 5 Jahren Honorare für Beratung und/oder Vorträge und/oder Sponsoring von MSD und Kyowa Kirin erhalten zu haben. K.G. gibt an, in den letzten 5 Jahren Honorare für Beratungen und/oder Vorträge und/oder Sponsoring wissenschaftlicher Projekte und/oder eine Mitwirkung als Studienarzt in klinischen Studien von beziehungsweise für folgende, für diese Arbeit relevante Firmen erhalten zu haben: AbbVie, Bristol Myers Squibb, LEO Pharma, Lilly, Pfizer, Incyte.

## CME Questions/Lernerfolgskontrolle

1. Welche Aussage ist richtig?
  - JAKi hemmen selektiver spezifische Zytokine als Biologika.
  - JAKi wirken, je nach Zieleinheit der JAK, immunmodulierend, antiproliferativ und entzündungshemmend.
  - Upadacitinib ist ein JAKi der ersten Generation.
  - Upadacitinib ist kein selektiver JAKi.
  - Tofacitinib ist zur Behandlung der Alopecia areata zugelassen.
2. Welche unerwünschte Wirkung/Erkrankung tritt am häufigsten als Nebenwirkung unter JAKi auf?
  - Myelosuppression
  - Herz‐Kreislauf‐Ereignisse
  - Malignome
  - Infektionen
  - Hepatotoxizität
3. Welche Aussage ist **falsch**?
  - JAKi können zum Anstieg der LDL‐Werte führen.
  - Unter Deucravacitinib sind Zytopenien nicht als Komplikation beschrieben.
  - Insbesondere JAK1 ist für die Aufrechterhaltung der Hämatopoese essenziell.
  - Ruxolitinib wird zur Behandlung myeloproliferativer Erkrankungen eingesetzt.
  - Unter oralen JAKi ist eine Kontrolle des Differenzialblutbildes im Verlauf empfohlen.
4. Welcher JAKi ist in der EU zur topischen Therapie des mittelschweren chronischen Handekzems des Erwachsenen, bei denen topische Kortikosteroide nicht ausreichen oder nicht geeignet sind, zugelassen?
  - Tofacitinib
  - Abrocitinib
  - Delgocitinib
  - Upadacitinib
  - Ruxolitinib
5. Welche Aussage ist richtig?
  - JAKi mit hepatischer Metabolisierung werden schneller ausgeschieden als JAKi mit renaler Metabolisierung.
  - Ruxolitinib ist in der EU der einzige zugelassene topische JAKi.
  - Die Sicherheit von JAKi in Schwangerschaft und Stillzeit ist durch Studien belegt.
  - JAKi sind nicht für die Therapie der Colitis ulcerosa zugelassen.
  - Akneiforme Hautveränderungen sind als dermatologische Nebenwirkungen unter JAKi nicht beschrieben.
6. Welcher JAKi kann laut Fachinformation in der Stillzeit angewandt werden?
  - Baricitinib, peroral
  - Abrocitinib, peroral
  - Deucravacitinib, peroral
  - Ruxolitinib, topisch
  - Delgocitinib, topisch
7. Welcher Parameter gehört **nicht** zum Sicherheitsmonitoring im Verlauf unter JAKi?
  - Kreatinin
  - γGT
  - Gesamtcholesterin
  - HIV‐Test
  - Anamnese zu klinischen Infektzeichen
8. Sie klären Ihren Patienten vor Therapieeinleitung über das erhöhte Risiko von Herpes zoster unter Upadacitinib auf. Wie viele Patienten unter Upadacitinib sind innerhalb eines Jahres davon betroffen?
  - Etwa jeder Zweite
  - Etwa jeder Dritte
  - Etwa jeder Vierte
  - Etwa jeder Zehnte
  - Etwa jeder Zwanzigste
9. Sie führen im Rahmen des Therapiemonitoring eine Laborkontrolle bei einem ihrer Patienten unter Upadacitinib vor. Bei welchem Parameter würden Sie die Therapie pausieren?
  - Hämoglobin 9,2 g/dl
  - Thrombozyten 193/nl
  - Neutrophile 1050/µl
  - Lymphozyten 442/µl
  - LDL‐Cholesterin 154 mg/dl
10. Sie beobachten in der Laborkontrolle Ihres Patienten mit Alopecia areata unter Baricitinib 4 mg 1 x täglich eine Anämie mit einem Hämoglobin von 7,9 g/dl. Sie haben kürzlich eine suffiziente Kontrolle der Krankheitsaktivität erreicht. Welche Option zur weiteren Behandlung wählen Sie?
  - Therapiefortsetzung
  - Dosissteigerung
  - Therapieunterbrechung
  - Dosisreduktion
  - Zusätzliche Eisensubstitution

Liebe Leserinnen und Leser, der Einsendeschluss an die DDA fur diese Ausgabe ist der 30. November 2025.

Die richtige Lösung zum Thema Supportive Schmerztherapie in der Dermatologie in Heft 06/2025 ist: 1a, 2c, 3e, 4d, 5a, 6a, 7c, 8c, 9c, 10e

Bitte verwenden Sie fur Ihre Einsendung das aktuelle Formblatt auf der folgenden Seite oder aber geben Sie Ihre Lösung online unter http://jddg.akademie-dda.de ein.
